# Well-defined tricobalt tetraoxide's critical morphology effect on the structure–reactivity relationship

**DOI:** 10.1039/d4ra02971b

**Published:** 2024-07-08

**Authors:** Sami Barkaoui, Noureddine Elboughdiri, Djamel Ghernaout, Yacine Benguerba

**Affiliations:** a Laboratoire Matériaux Traitement et Analyse, National Research Institute of Physical and Chemical Analysis, Technological Pole Sidi Thabet 2020 Sidi Thabet Tunisia samibarkaoui501@gmail.com; b Chemical Engineering Process Department, National School of Engineering Gabes, University of Gabes Gabes 6011 Tunisia ghilaninouri@yahoo.fr; c Chemical Engineering Department, College of Engineering, University of Ha'il PO Box 2440 Ha'il 81441 Saudi Arabia; d Chemical Engineering Department, Faculty of Engineering, University of Blida PO Box 270 Blida 09000 Algeria djamel_andalus@yahoo.fr; e Laboratoire de Biopharmacie et Pharmacotechnie (LBPT), Université Ferhat ABBAS Sétif-1 Sétif Algeria benguerbayacine@yahoo.fr

## Abstract

This review focuses on exploring the intricate relationship between the catalyst particle size and shape on a nanoscale level and how it affects the performance of reactions. Drawing from decades of research, valuable insights have been gained. Intentionally shaping catalyst particles makes exposing a more significant percentage of reactive facets possible, enabling the control of overactive sites. In this study, the effectiveness of Co_3_O_4_ nanoparticles (NPs) with nanometric size as a catalyst is examined, with a particular emphasis on the coordination patterns between oxygen and cobalt atoms on the surface of these NPs. Investigating the correlation between the structure and reactivity of the exposed NPs reveals that the form of Co_3_O_4_ with nanometric size can be modified to tune its catalytic capabilities finely. Morphology-dependent nanocatalysis is often attributed to the advantageous exposure of reactive crystal facets accumulating numerous active sites. However, experimental evidences highlight the importance of considering the reorganization of NPs throughout their actions and the potential synergistic effects between nearby reactive and less-active aspects. Despite the significant role played by the atomic structure of Co_3_O_4_ NPs with nanometric size, limited attention has been given to this aspect due to challenges in high-resolution characterizations. To bridge this gap, this review strongly advocates for a comprehensive understanding of the relationship between the structure and reactivity through real-time observation of individual NPs during the operation. Proposed techniques enable the assessment of dimensions, configuration, and interfacial arrangement, along with the monitoring of structural alterations caused by fluctuating temperature and gaseous conditions. Integrating this live data with spectroscopic methods commonly employed in studying inactive catalysts holds the potential for an enhanced understanding of the fundamental active sites and the dynamic behavior exhibited in catalytic settings.

## Abstract 2

This review focuses on exploring the intricate relationship between the catalyst particle size and shape on a nanoscale level and how it affects the performance of reactions.

## Graphical abstract

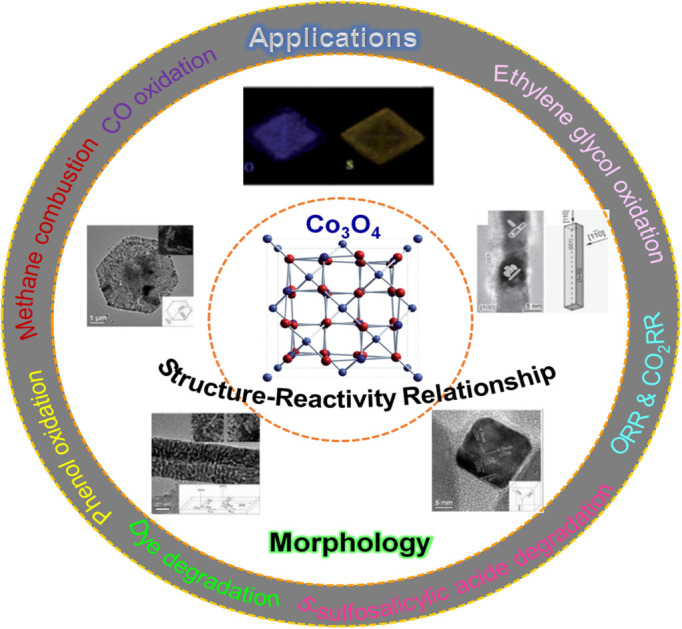

## 1. Introduction

A catalyst's principal role is to facilitate the acceleration of a given process without compromising the reaction's integrity. Pt-group metals such as Ir, Ru, Pt, and Pd are acknowledged for their exceptional catalytic activity.^1,2^ However, their high cost and scarcity on Earth have motivated scholars to investigate cost-effective alternatives with an abundance on the planet.^3,4^ Transition metals including cobalt (Co), Fe, and Ni have surfaced as viable substitutes for catalysts based on platinum group metals.^5–8^ First-row transition metals such as Co exhibit diverse properties, acting as electron inks or sources, existing in various oxidation states, and participating in electron exchange.^9–12^ With its three unoccupied d orbitals, cobalt bonds with surface-bound chemical species, enhancing catalytic activity, especially structural flaws such as vacancies near the crystal lattice surface.^13^ Cobalt-based catalysts including tricobalt tetraoxide (Co_3_O_4_) have gained significant attention in Europe for their extensive use in energy and environmental industries. The catalytic properties of Co, attributed to its partly filled d orbital (3d^7^), allow for facile composite creation by combining it with other elements or supports. Co and Co-based nanostructures have been investigated to enhance the surface area of catalysts, therefore exposing a more significant number of active sites and allowing the selective exposure of the most active catalytic centers. Co's ability to transition between the Co^2+^ and Co^3+^ oxidation states based on redox conditions makes it an ideal reagent complex builder.^14^

Cobalt's dual oxidation state takes advantage of surplus electrons during a reaction, demonstrating its adaptability. The spinel crystal structure of Co_3_O_4_ contributes to its multifunctional semiconductor properties, and Co_3_O_4_ nanoparticles (NPs) exhibit direct optical band gaps, making them suitable for visible light photocatalysis.^15,16^ Co_3_O_4_'s varied spin states, such as high, low, and intermediate spin, make it intriguing from a fundamental and spintronic perspective.^16^ Cobalt's versatility extends to its environmental impact, as demonstrated by Co_3_O_4_'s ability to oxidize various compounds, including carbon monoxide (CO), volatile organic compounds, sulfur dioxide (SO_2_), and hydrocarbons. Co_3_O_4_ is employed in processes such as three-way catalytic conversion, phenol oxidation, diesel soot oxidation, and clean energy production, such as hydrogen through steam reforming methanol and ethanol.^17–20^ Additionally, Co_3_O_4_ serves as a commercial catalyst in the oxidation, hydrogenation, and hydrogenolysis of esters.^21^

This review examines recent advancements in the shape engineering of Co_3_O_4_ with nanometric size. It focuses on their catalytic performance, which is influenced by the coordination patterns of oxygen and cobalt atoms on their surface. The analysis encompasses progress in this field, exploring the structure–reactivity relationship concerning exposed NPs. The review concludes with a summary and a perspective on future developments, aiming to inform readers about the potential prospects involving Co_3_O_4_-based catalysts.

## 2. Preparation strategy of the morphological Co_3_O_4_

Much effort has been dedicated to preparing Co_3_O_4_ with well-controlled shapes, sizes, and crystal structures. Co_3_O_4_ has been engineered into zero-dimensional (0D) NPs,^22^ one-dimensional (1D) structures such as nanorods (NRs),^23^ nanowires,^24,25^ two-dimensional (2D) nanodiscs, or nanosheets,^26–29^ three-dimensional (3D) nanocubes (NCs),^30,31^ and even hierarchical nanoflowers or more complex structures.^32–34^ Some of the more well-liked methods to form these nanostructures are coprecipitation, ultrasonic spray pyrolysis, thermal decomposition, microwave-assisted, hydrothermal, and solvothermal methods are examples of physical and chemical processes that have been used for the preparation of Co_3_O_4_ with nanometric size.^35–40^ NPs enclose outstanding features, such as a simple and economical synthesis method, high surface area, good stability, and uncomplicated recovery. These properties put together more approval than other synthesis strategies of the prepared catalysts. Researchers have tried to prepare Co_3_O_4_ with nanometric size by different shapes using different methods to obtain a cost-effective, simple procedure, shorter time through an effective manner, and rectify the purity of the synthesized prepared sample. These processes include.

### 2.1. Coprecipitation

The coprecipitation method is a simple, efficient, and economical method for the mass production of ultrafine nano-powders. Homogeneity, purity, and reactivity of the prepared oxide are the other advantages of this method. This method was used to prepare Co_3_O_4_ with nanometric size.^35^ First, Co(NO_3_)_2_·6H_2_O was dissolved in deionized water. Secondly, ammonium oxalate was added to the solution with continuous stirring. The precipitate was then washed with deionized water and dried at room temperature. Finally, it was calcined at 400–500 °C for 3 h. The average size of the obtained NPs was from 40 to 350 nm, and the Co_3_O_4_ NPs have an average diameter of 100 nm.

### 2.2. Utrasonic spray pyrolysis

Ultrasonic spray pyrolysis is an efficient, controlled, and versatile synthesis method. It is frequently used to prepare transition metal oxides, particularly Co_3_O_4_,^36^ through high purity and narrow size distribution.

Three different precursor solutions were prepared by dissolving cobalt acetate, cobalt chloride, or cobalt nitrate in distilled water with a concentration of cobalt salt as 0.5 mol L^−1^.^36^ The starting solution was aerosolized using an ultrasonic nebulizer (Omron, model NB-150U) with a frequency of 1.75 MHz. The spray pyrolysis temperature was kept at 750 °C. The obtained powders were collected at the reactor exit. The prepared Co_3_O_4_ samples from cobalt acetate, cobalt chloride, and cobalt nitrate are denoted as A–Co_3_O_4_, C–Co_3_O_4_, and N–Co_3_O_4_. According to the X-ray diffraction (XRD) data of A–Co_3_O_4_, C–Co_3_O_4_, and N–Co_3_O_4_ samples, all the prepared samples adopted a spinel-type cubic structure. The characteristic diffraction peaks are sharp, and no impurities or a second phase were detected, affirming that high-purity Co_3_O_4_ was obtained. Scanning electron microscopy (SEM) was used to examine the shapes of the A–Co_3_O_4_, C–Co_3_O_4_, and N–Co_3_O_4_ samples. For the A–Co_3_O_4_ powders, the dimple and wrinkle surface can be observed. C–Co_3_O_4_ sample has a porous spherical morphology, and microspheres are developed from various closely packed primary particles; moreover, abundant voids are left among adjacent particles. The N–Co_3_O_4_ sample has a durian-like shape with a 0.5–3 μm size distribution, suggesting a hollow inner structure.

### 2.3. Thermal decomposition

The thermal decomposition of metal oxides performed in high boiling point organic solvents and the existence of surfactants are highly relevant. This process is mainly recognized for preparing excellent-quality NPs with small sizes, high crystallinity, and narrow particle size distributions, although the resulting NPs are very stable in organic solvents.^41–45^ Nonetheless, this approach has some associated drawbacks, *e.g.*, it requires preparation at high reaction temperatures, an inert atmosphere, and long processing times, resulting in increased energy and time consumption.

For example, cobalt oxalate was used as a precursor for synthesizing Co_3_O_4_NRs by thermal decomposition.^37^ 0.6 g of cobalt oxalate and 5 mL oleylamine (as a surfactant) were placed in a 50 mL two-neck distillation flask and heated up to 140 °C for 1 h. The resulting solution was added to 5 g of triphenylphosphine (as a surfactant) at 240 °C. The black solution was maintained under stirring at 240 °C for 45 min and then cooled to room temperature. The final sample was washed with ethanol several times to remove the excessive surfactant. Transmission electron microscopy (TEM) was used to verify the size and shape of the prepared samples. The TEM images of Co_3_O_4_NRs demonstrated that the materials had rod-like shapes. The length of NRs was 400–550 nm, and their diameters were about 20 nm.

### 2.4. Microwave-assisted methods

Microwave-assisted chemistry is becoming essential in every area of synthetic chemistry since it can boost some competitive advantages over other preparation methods. It could reduce the processing times and enhance the crystallization level of the particles. These advantages of microwave–hydrothermal methods over conventional hydrothermal methods arise from the direct interaction of the microwaves with the ions or molecules in the solution and with the solid phases dispersed in the liquid medium. In effect, it is essential to underline that the efficiency in the conversion capacity of microwave energy into thermal energy is governed by the physics variables: loss tangent, relaxation time, and penetration depth.^46^ Non-aqueous solvents (glycerol, ethylene glycol (EG), propylene glycol) have been frequently used^47,48^ to avoid or minimize the agglomeration process between the particles.

This method produces high yields, simple to operate, and efficient in terms of being environmentally friendly and energy-consuming. Also, it has been extensively applied to prepare inorganic nanostructured materials^49–56^ with applications, *e.g.*, electrodes,^57^ humidity sensors,^58^ or catalytic devices.^52^ The method's versatility for synthesizing NPs has been especially reported.^59^ The microwave-assisted hydrothermal route has been developed to prepare Co_3_O_4_ with NRs' shape.^40^ The method involved two steps: first, NRs of cobalt hydroxide carbonate were prepared by mixing 50 mL of 0.6 M Co(NO_3_)_2_·6H_2_O and 2.4 g of CO(NH_2_)_2_ under 500 W microwave irradiated for 3 min. Subsequently, the cobalt hydroxide carbonate NRs were calcined under air at 400 °C for 3 h to fabricate Co_3_O_4_NRs. After the thermal decomposition of cobalt hydroxide carbonate precursor under 400 °C for three hours, a single phase of well-crystallized Co_3_O_4_ with the cubic structure was obtained, and no peaks of the other phase were detected, indicating that the sample was of high purity. The as-prepared sample was bamboo-like NRs with a diameter varying from 30 to 60 nm and a length of 100 to 1000 nm.

### 2.5. Hydrothermal and solvothermal methods

The hydrothermal method is one of the best-used processes for preparing nanomaterials. It is essentially a solution reaction-based approach. To control the shape of the prepared materials, either low-pressure or high-pressure conditions can be used depending on the vapor pressure of the main composition in the reaction. It has numerous advantages over the other conventional methods such as energy saving, simplicity, cost-effectiveness, acceleration interaction between solid and species, better nucleation control, higher dispersion, pollution-free (as the reaction is done in a closed system), higher rate of the reaction, and lower temperature of operation in the presence of a suitable solvent. Also, it provides highly crystalline particles with better control over their size and shape.

The solvothermal process is similar in its technology to the hydrothermal one, as it is carried out in autoclaves at high temperatures and pressure, through just one difference: instead of water, the synthesis is carried out in organic solvents. Co_3_O_4_ nanostructures with different morphologies (NCs, nanowires, nanobundles, nanoplates (NLs), and nanoflowers) have been prepared,^38,52^ and the experimental details of the preparation of Co_3_O_4_ nanostructures with different shapes are summarized in Table 1.

**Table 1:** Experimental parameters of the preparation of different shapes of Co_3_O_4_ nanostructures

Shape	Cobalt salt (mM)	Temperature (°C)	Reaction time (h)	Structure-directing agents
Nanocubes (NCs)	2	180	12	15 mL of ammonia (6%)
Nanowires	2	150	5	30 mL ethanol (99.9%) and 3 mmol of urea
Nanobundles	2	120	12	2 mmol urea
Nanoplates (NLs)	2	150	15	3 mL NaOH solution (3.25 mM) with 2 mL ammonia (6%)
Nanoflowers	2	180	12	30 mL ethanol and 15 mL ammonia (6%)

## 3. Tuning the morphology of Co_3_O_4_ for the catalytic reaction

**Fig. 1:**
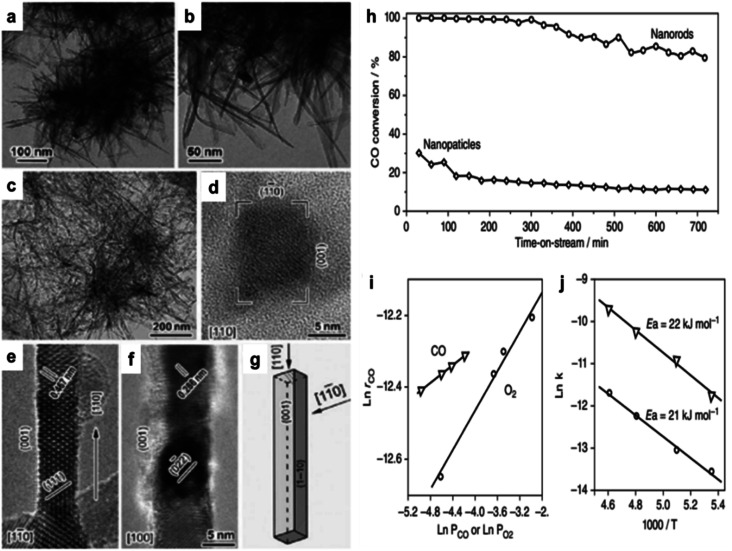
Transmission electron microscope (TEM) and high-resolution transmission electron microscope (HRTEM) images of cobalt-based nanostructures: (a, and b) cobalt hydroxide carbonate; (c–f) Co_3_O_4_ nanorods (NRs), (c) low-magnification bright-field view, and (d–f) high-resolution views at {110}, {1–10}, and {100}; (g) NR morphological illustration. Catalytic performance of Co_3_O_4_: (h) CO conversion efficiency over time for Co_3_O_4_ nanoparticles (NPs) and NRs in a continuous-flow reactor at −77 °C; (i) reaction rate (*r*_CO_) *vs.* CO or O_2_ concentrations for Co_3_O_4_ NRs; (j) Arrhenius plots (based on ref. 64, Copyright 2009, Nature Publishing Group).

Cobalt oxide is used mainly as a catalyst. Co_3_O_4_ was used as a model in oxidizing CO.^60–65^ An initial investigation revealed that Co_3_O_4_ might facilitate the oxidation of CO at temperatures as low as −54 °C. The activity was significantly decreased, however, when the reaction gas included trace amounts of moisture (3–10 ppm), which obscured the active Co^3+^ sites.^66,67^ Cobalt oxide's activity and durability in the CO oxidation process were increased by changing its form from spherical NPs to NRs, demonstrating a solid morphology-dependent impact.^64^ NR-shaped cobalt hydroxycarbonate was generated by precipitating cobalt acetate with sodium carbonate in EG. As seen in Fig. 1a–c, further calcination at 450 °C in air converted this precursor into rod-shaped Co_3_O_4_ NR measuring 200–300 nm in length and 10–20 nm in diameter. The CO oxidation method using spherical NPs yielded an initial CO conversion of 30% at −77 °C. However, as the time-on-stream increased, this conversion decreased to around 10% (Fig. 1h). More active and stable than Co_3_O_4_ NP catalysts, NR catalysts demonstrated 100% CO conversion in the first 6 h and maintained an 80% CO conversion for ∼12 h after the reaction.

In contrast to the spherical NPs, Co_3_O_4_ NR demonstrated an approximately one-order-of-magnitude increase in the rate of CO oxidation. At −77 °C, the Co_3_O_4_ NR reaction rate was 3.91 × 10^−6^ mol_CO_ g^−1^ s^−1^. Conversely, the value of the NPs was just 4.66 × 10^−7^ mol_CO_ g^−1^ s^−1^. The high-resolution transmission electron microscope (HRTEM) analysis revealed that the Co_3_O_4_ NPs were enclosed by a configuration consisting of eight {111} and six {001} planes. Conversely, the Co_3_O_4_ NR preferred to reveal the {110} planes, constituting an estimated 40% of their overall surface area (Fig. 1g). It was found that Co^3+^ species functioned as active sites for CO oxidation on the {110} plane.

The performance of Co_3_O_4_ nanobelts (NBs) and NCs in CO oxidation has been investigated.^63^ The reaction rate of Co_3_O_4_ NC, which mostly exposed the ∼{001} facets, was 0.62 μmol g^−1^ s^−1^, as opposed to the 0.85 μmol g^−1^ s^−1^ seen on NBs terminated by the {110} plane. The specific conversion rate indicates that at 56 °C, Co_3_O_4_ NB exhibits 1.37 times the activity of CO_3_O_4_ NCs, demonstrating that the Co_3_O_4_ NB are significantly more active than Co_3_O_4_ NC. As shown by these studies, the activation of the surface layer lattice oxygen on the {110} planes is more pronounced in the presence of Co^3+^ species compared to the {001} planes. Furthermore, it was shown that Co_3_O_4_ nanowires (NWs) enclosed in {111} planes and measuring around 3 nm in diameter had a notably increased rate of CO oxidation at 248 °C, amounting to 161.75 μmol CO g^−1^ s^−1^.^61^ The enhanced performance resulted from the increased surface area and the profusion of Co^3+^ cations on the surfaces.

**Fig. 2:**
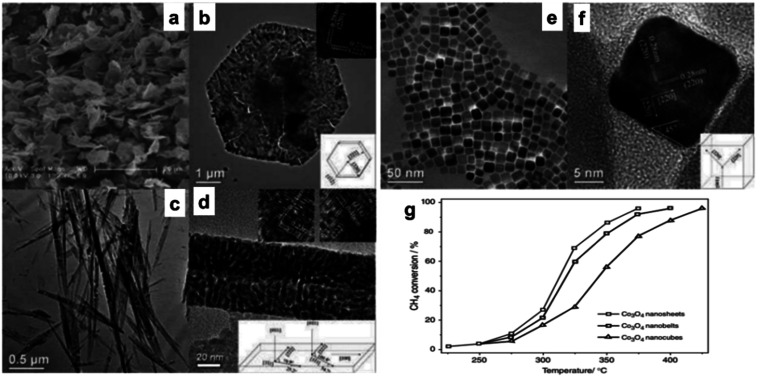
Scanning electron microscopy (SEM) and high-resolution transmission electron microscope (HRTEM) analysis with structural models of Co_3_O_4_ nanostructures: (a and b) Co_3_O_4_NS; (c and d) Co_3_O_4_NB; (e and f) Co_3_O_4_NC. (g) Methane conversion efficiency *vs.* temperature for Co_3_O_4_ at a GHSV of 40 000 h^−1^ (based on ref. 30, Copyright 2008, American Chemical Society).

A catalytic study for CO oxidation^62^ indicates that Co_3_O_4_ NR exposed to {111} planes exhibited enhanced activity at an activation energy of 40 kJ mol^−1^, whereas Co_3_O_4_NLs exposed to the same planes had superior activity at a reduced activation energy of 21 kJ mol^−1^. Significant morphology-dependent effects on CO oxidation have been observed, contradicting prior hypotheses to some degree (maybe due to the porous structures amid cracks and interspaces in the Co_3_O_4_ nanostructures). The formation of Co_3_O_4_ NS, Co_3_O_4_ NB, and Co_3_O_4_ NC by hydrothermal synthesis of a cobalt hydroxide precursor followed by direct thermal breakdown was investigated in kinetic experiments for methane (CH_4_) combustion (Fig. 2a–f).^30^ The specific rates (*r*_CH4_) for Co_3_O_4_ NC (343 °C), Co_3_O_4_ NB (319 °C), and Co_3_O_4_ NS (313 °C) were 1.25, 2.28, and 2.72 μmol g^−1^, respectively, as shown in Fig. 2g. Additionally, the *T*_50_, representing the temperature at which half of the methane conversion occurred, exhibited a decreasing trend in the same sequence. The structural study indicated that the most prevalent planes on Co_3_O_4_ NS, Co_3_O_4_ NB, and Co_3_O_4_ NC were {112}, {110}, and {001}, respectively. Beyond these crystal planes, the methane combustion process persisted in the following order: {112} > {110} ⟫ {001}. It can be deduced that manipulating the structure of nanostructured cobalt oxides leads to a substantial display of catalytically active sites. This is supported by the enhanced CH_4_ combustion activity observed in Co_3_O_4_ as a nanosheet, which exposes the more reactive {112} planes. The catalytic activity of Co_3_O_4_ supported on stainless steel wire mesh, produced by the ammonia evaporation process, was investigated with the preferred oxidation (PROX) of CO.^68^ The 500 nm-diameter mesoporous Co_3_O_4_ nanowires' diameter is 3.4 nm, and they have a Brunauer–Emmett–Teller (BET) surface area of 71 m^2^ g^−1^. This structured catalytic system is very stable over the whole temperature range of 100–175 °C due to its low-pressure drop and high heat exchange rate; furthermore, its exceptional catalytic activity is twice that of the highest-performing Co_3_O_4_ catalyst previously documented.

**Fig. 3:**
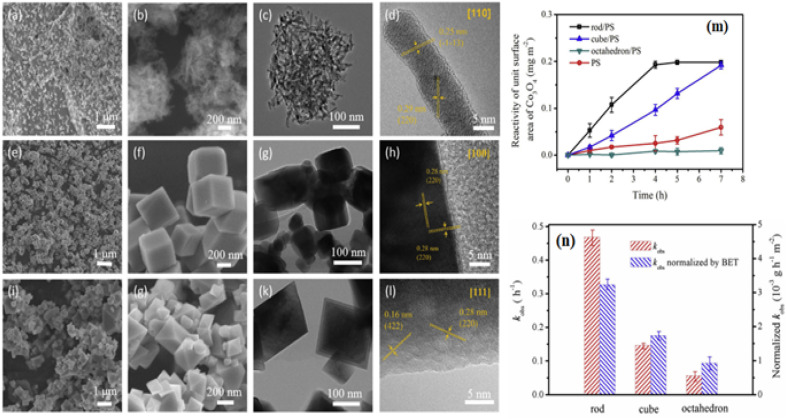
Morphological and catalytic characteristics of Co_3_O_4_ nanostructures: scanning electron microscopy (SEM) and high-resolution transmission electron microscope (HRTEM) images of (a–d) Co_3_O_4_NR {110}, (e–h) Co_3_O_4_NC {100}, and (i–l) Octahedra {111}; (m) phenol oxidation reaction rates using peroxydisulfate and Co_3_O_4_ at pH 11; (n) comparison of rate constants and Brunauer–Emmett–Teller (BET)-normalized rate constants for different Co_3_O_4_ facets (adapted from ref. 16, © 2020 Elsevier Ltd).

Although PROX was believed to have an active Co^3+^ site, its mechanism may have been distinct from the low-temperature oxidation of the CO reaction. Researchers^69^ stated that the turnover frequency of Co_3_O_4_ NC, composed of six 100-facet facets, was 3.5 to 4 times more than that of Co_3_O_4_ NS, Co_3_O_4_ NB, and Co_3_O_4_ NP. Besides reducing Co^2+^ in hydrogen-rich environments, spectroscopic investigations revealed that Co_3_O_4_ NC's bulk and surface Co^3+^ sites were only modestly stabilized. For selective CO oxidation, the optimum pair Co^3+^/Co^2+^ was used. By using a sequence of Co_3_O_4_ catalysts, including exposed {111}, {110}, and {100} planes, it was verified that Co^3+^ functioned as the active site. As shown from the linear relationship between the number of Co^3+^ surface areas and the quantity of CO_2_ produced,^70^ the 100 facets positively impacted the PROX. Analyses of different Co_3_O_4_ attributes indicate that the phase and surface characteristics, including shape, surface area, and facets, significantly affect the catalytic activity. As seen in Fig. 3a–l,^16^ the synthesis of Co_3_O_4_, including a variety of Co_3_O_4_ NR {110}, Co_3_O_4_ NC {100}, and nano-octahedron {111} (NO) facets has been completed. The catalytic reactivity of Co_3_O_4_ NR, Co_3_O_4_ NC, and Co_3_O_4_ NO was the highest for phenol oxidation by the persulfate (PS) process. Fig. 3m and n demonstrated that the Co_3_O_4_ NR exhibited the lowest adsorption energy estimated by the density functional theory (DFT). This confirms that PS is more easily activated *via* a non-radical pathway on the Co_3_O_4_ {110} plane.^16^

**Fig. 4:**
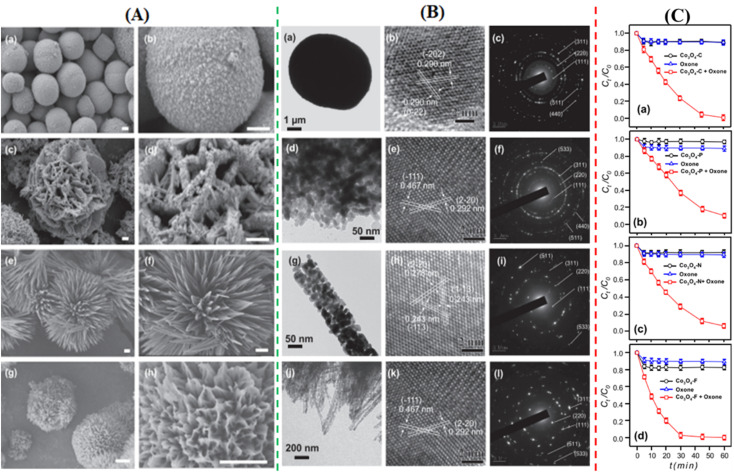
Morphological analysis of 3D Co_3_O_4_: (A) scanning electron microscopy (SEM) images of (a and b) Co_3_O_4_NC, (c and d) Co_3_O_4_nanoplates (NLs), (e and f) Co_3_O_4_NN, and (g and h) Co_3_O_4_NF. (B) Transmission electron microscope (TEM) images with electron diffraction patterns of (a–c) Co_3_O_4_NC, (d–f) Co_3_O_4_NLs, (g–i) Co_3_O_4_NN, and (j–l) Co_3_O_4_NF. (C) Comparative analysis of Co_3_O_4_ catalysts in oxone activation for 5-sulfosalicylic acid degradation of (a) Co_3_O_4_NC, (b) Co_3_O_4_NLs, (c) Co_3_O_4_NN, and (d) Co_3_O_4_NF (adapted from ref. 71, © 2020 Elsevier BV).

**Fig. 5:**
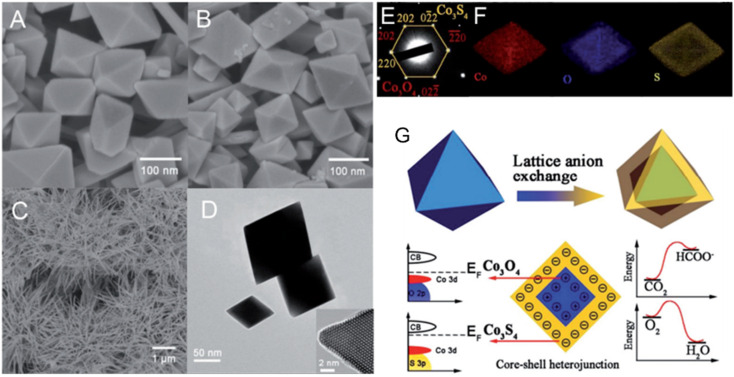
Structural and compositional analysis of Co_3_O_4_ and Co_3_S_4_ nanostructures: (A) FE-scanning electron microscopy (FE-SEM) image of Co_3_O_4_NO; (B) Co_3_S_4_@Co_3_O_4_NO; (C) Co_3_S_4_ nanoneedles. (D) Transmission electron microscope (TEM) and high-resolution transmission electron microscope (HRTEM) images of Co_3_S_4_@Co_3_O_4_. (E) Selected area electron diffraction pattern of Co_3_S_4_@Co_3_O_4_. (F) EDX elemental mapping of Co_3_S_4_@Co_3_O_4_. (G) Schematic of heterojunction-assisted Co_3_S_4_@Co_3_O_4_ for oxygen reduction reaction (ORR) and CO_2_ reduction reaction (CRR) (adapted from ref. 72, © 2017 WILEY-VCH Verlag GmbH & Co. KGaA, Weinheim).

To degrade 5-sulfosalicylic acid, four distinct 3D Co_3_O_4_ catalysts were fabricated, each with a unique morphology (Fig. 4): Co_3_O_4_ NC {111}, Co_3_O_4_ NLs {110}, Co_3_O_4_ NNs (nanoneedles, {110}), and Co_3_O_4_NFs (nanoflowers, {112}).^71^ Primarily, Co_3_O_4_ NF ({112} facets) is the most beneficial 3D Co_3_O_4_ catalyst for the oxidation activation to degrade 5-sulfosalicylic acid^71^ due to its plentiful Co^2+^ and more reactive surface, in addition to its most excellent surface area (121.1 m^2^ g^−1^). The core–shell contrast ratio of the as-prepared Co_3_S_4_@Co_3_O_4_ core–shell octahedron catalyst *via* hydrothermal and post-surface lattice anion exchange is comparatively less than that of the other core–shell structures.^72^ This is because the concentrations of Co_3_S_4_ and Co_3_O_4_ are close. The hexagonal shape of the selected area electron diffraction pattern, as seen in Fig. 5E, corresponds to both the {111} facet exposure and the close-packed hexagonal pattern observed in the inset of Fig. 5D in HRTEM. The lattice spacing of the {220} pattern is 0.33 nm. As seen in Fig. 5G, electrochemical CO_2_ reduction reaction (CRR) and oxygen reduction reaction (ORR) were investigated using a core–shell configuration of Co_3_O_4_ NO coated with a Co_3_S_4_ surface. A distinctive electronic configuration is bestowed by the heterojunction separating the p-type Co_3_O_4_ core and the n-type Co_3_S_4_ shell, enabling both catalytic processes.

**Fig. 6:**
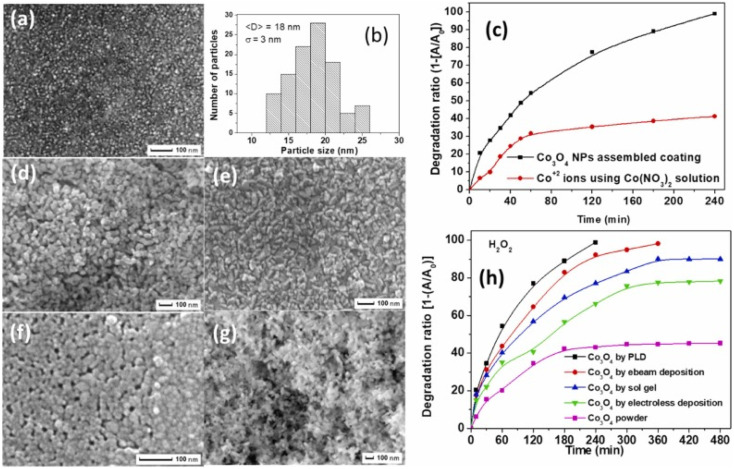
Co_3_O_4_ catalysts synthesized *via* various methods and their photocatalytic performance: (a) SEM images of coatings prepared by PLD, (b) particle size distribution histogram for PLD coatings, (c) time-dependent photocatalytic degradation of MB using Co_3_O_4_ NPs assembled coating *via* PLD and cobalt nitrate and (d–g) SEM images respectively of coatings prepared by (d) electroless, (e) electron beam, (f) sol–gel depositions, and powder form; (h) comparative photocatalytic efficiency of powder Co_3_O_4_ and coatings by different methods (adapted from ref. 73 and 74 © 2012 Elsevier BV).

To solve the recovery issue and make a reusable, eco-friendly “green” catalyst, the optimum catalyst is Co_3_O_4_ with nanometric size attached to a particular substrate with solid adhesion. Chemical (sol–gel), physical (pulsed laser deposition, or PLD), and electrochemical (electroless) methods have been used to create coatings that are reconstructed with Co_3_O_4_ with nanometric size. Fig. 6a and b shows that the Co_3_O_4_ NPs generated using the PLD approach without post-annealing treatment have a mixed amorphous–nanocrystalline phase, a tiny average size of 18 nm, a narrow size distribution of *σ* = 3 nm, a perfectly spherical form, and allow a degree of accumulation.^73,74^

In a methylene blue (MB) solution, the activity of a homogeneous catalyst generating Co^2+^ ions was compared to that of a thin coating catalyst constructed with heterogeneous Co_3_O_4_ with nanometric size. Complete mineralization of MB dye was achieved in 240 min, indicating a far greater degradation rate than the 40% removed by Co^2+^ ions (Fig. 6c). In the same study, researchers^73^ found that coatings made of assembled Co_3_O_4_ with nanometric size had a slightly lower catalytic activity but still demonstrated good recycling capability. Fig. 6d–g shows that PLD-deposited Co_3_O_4_ coatings have the superior photo-degradation rate of MB dye when compared to Co_3_O_4_ coatings made using other processes (*i.e.*, electro-beam deposition, sol–gel, and electroless) that have almost equal particle-like morphology (Fig. 6h).

## 4. Co_3_O_4_-supported metal nanoparticles (NPs)

**Fig. 7:**
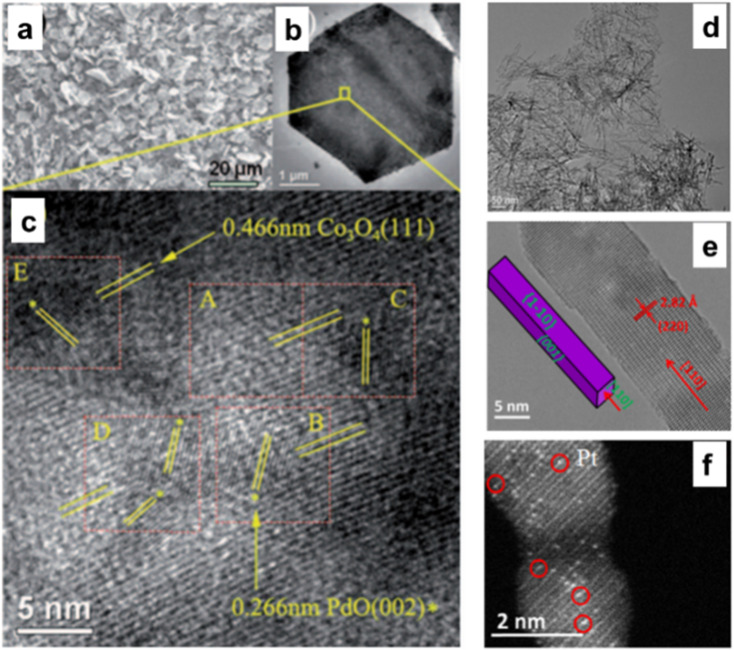
Microscopic analysis of 5% Pd-doped Co_3_O_4_NS: (a) scanning electron microscopy (SEM), (b) transmission electron microscope (TEM), and (c) high-resolution transmission electron microscope (HRTEM) images highlighting of PdO {002} and 0.466 nm of Co_3_O_4_ {111} (reproduced with permission from ref. 75, Copyright 2011, WILEY-VCH Verlag Gmbh& Co). Detailed TEM and scanning transmission electron microscopy (STEM) analysis of Co_3_O_4_NR catalysts: (d) TEM, (e) HRTEM, and (f) STEM image of Pt atoms singularly dispersed on Co_3_O_4_NR (reproduced with Permission from ref. 77, Copyright; American Chemical Society).

Co_3_O_4_ has been considered an active support for heterogeneous catalysis for a very long time and is distinguished by its solid metal support interactions. As stated, Co_3_O_4_ with nanometric size has an evident morphological influence on CH_4_ combustion in the following sequence: Co_3_O_4_ NS, Co_3_O_4_ NB, and Co_3_O_4_ NC.^30^ Despite applying the same quantity of Pd NPs to these materials, the Pd/Co_3_O_4_ NS catalyst continued to produce the most methane combustion. The PdO {111} and Co_3_O_4_ NS formed a geometrically advantageous match, particularly on the {112} facet (Fig. 7a–c), which enhanced the solid metal support interactions and subsequently facilitated the activation of C–H bonds.^75^ The number of missing neighbors of a Co_3_O_4_ unit cell on a plane {112} is five for the NS shape. PdO must be sited in the 5-fold center of the surface of Co_3_O_4_ NS as a thin discrete film through a matching geometry and strong coordination rather than a top or bridge site.^76^

Due to its low activation barrier of 29.6 kJ mol^−1^, single Pt atoms attached to Co_3_O_4_ demonstrate significant catalytic activity in the water gas shift process at 200 °C (turnover frequency = 0.58 mol_H2_ per site_Pt_ per s). The significantly decreased activation energy observed for these individual Pt atoms may indicate that the interaction between the atoms and the 110-faced Co_3_O_4_ NR substantially customized the chemical environment of the active sites (Fig. 7d–f).^77^

**Fig. 8:**
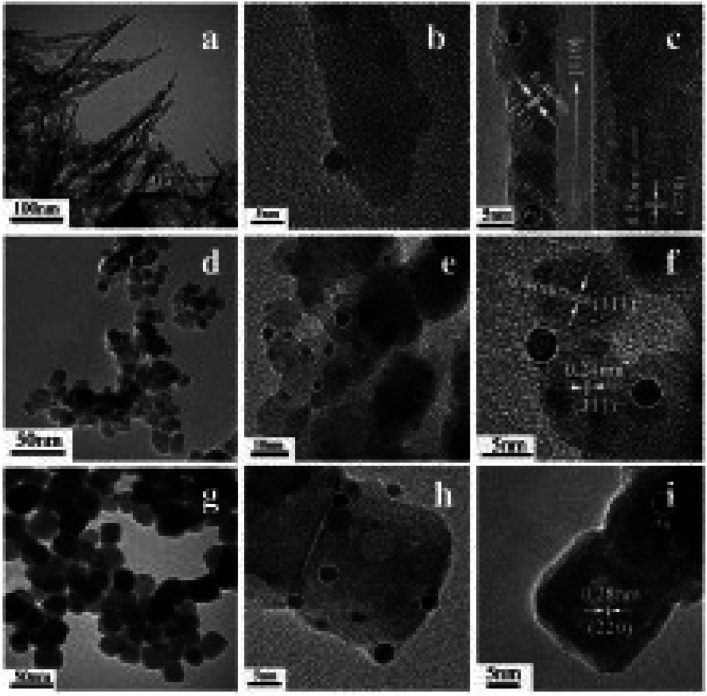
Transmission electron microscope (TEM) images of Co_3_O_4_NR (a), Co_3_O_4_NH (d), and Co_3_O_4_NC (g). High-resolution transmission electron microscope (HRTEM) images of Au/Co_3_O_4_NR (b, and c), Au/Co_3_O_4_NH (e, and f), and Au/Co_3_O_4_NC (h, and i). Source: reprinted with permission from ref. 78, ©2011 Elsevier BV.

Using straightforward hydrothermal and solvothermal techniques, anion adsorption was employed to deposit gold NPs onto Co_3_O_4_ materials produced in various forms, including rods, polyhedra, and cubes.^78^ Au catalysts based on Co_3_O_4_ were characterized using TEM and HRTEM. The predicted morphologies of the Co_3_O_4_ supports are cube-shaped, rod-shaped, and polyhedron (NH)-shaped (Fig. 8). Research into the exposed planes of various morphological Co_3_O_4_ materials has led to the discovery that the morphology of the support plays a crucial role in determining the catalytic activity. Co_3_O_4_ NR shows {110} planes most of the time on HRTEM, whereas the {011} and {001} planes are the most prominent on Co_3_O_4_ NH and Co_3_O_4_ NC structures, respectively. The {110} plane has the most excellent oxygen vacancies, which are very important for the oxidation of ethylene, in comparison to the {011} and {001} planes. Consequently, the ethylene conversion rate of 93.7% was achieved by Au/Co_3_O_4_ NR, demonstrating their exceptional catalytic activity. Ethanol conversion was 85.5% for the Au/Co_3_O_4_ NH catalyst. At 0 °C, the ethylene conversion on Au/Co_3_O_4_ NC was 26.8%, which was the lowest value recorded.

**Fig. 9:**
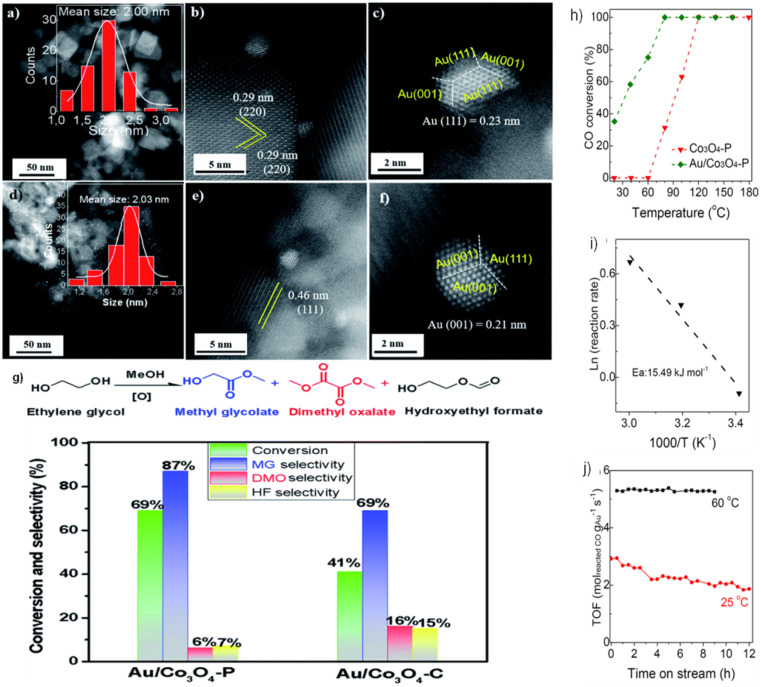
Scanning transmission electron microscopy (STEM) imaging and catalytic performance of Au-doped Co_3_O_4_: (a–c) Au particles on Co_3_O_4_ {001} in Au/Co_3_O_4_NC; (d–f) Au particles on Co_3_O_4_ {111} in Au/Co_3_O_4_ nanoparticles (NPs); (g) catalytic efficiency of Au/Co_3_O_4_NPs and Au/Co_3_O_4_NC in ethylene glycol (EG) oxidation. Reprinted with Permission from ref. 79, 2021 Royal Society of Chemistry. Performance analysis of Co_3_O_4_ and Au/Co_3_O_4_ in CO oxidation: (h) temperature-dependent catalytic activity for CO oxidation; (i) Arrhenius plots showing rate *vs.* 1/*T* for CO oxidation over Au/Co_3_O_4_; (j) durability tests at 25 °C and 60 °C with CO conversion rates from 25% to 45%. Reprinted with Permission from ref. 80, 2023 Royal Society of Chemistry.

Our prior research^79^ examined the effect of Co_3_O_4_ crystallization on EG oxidation supports in the form of Co_3_O_4_ NCs and NLs. As shown in Fig. 9a–c, Au NPs in the Au/Co_3_O_4_ NCs samples exhibited a quasi-truncated octahedron structure with Au {111} and {100} faces and had an average size of 2.0 nm. As shown by the interplanar distance of 0.29 nm, corresponding to the {220} crystal plane of cubic Co_3_O_4_ oxides, Au NPs are anchored consistently on the Co_3_O_4_ {001} facet. Furthermore, the inter-planar spacing of 0.46 nm corresponds to the lattice fringes seen in Au/Co_3_O_4_ NL catalysts and is caused by the Co_3_O_4_{111} facets of Co_3_O_4_ NL oxides. The uniform loading of Au particles onto the Co_3_O_4_ {111} facet resulted in the formation of a quasi-truncated octahedron encircled by Au {111} and {100} facets, as seen in Fig. 9d–f. Under these conditions, the Co_3_O_4_ NC and Co_3_O_4_ NL constituents remained dormant during the aerobic oxidation of EG. With the addition of Au NPs, the catalytic activity of EG oxidation processes was substantially enhanced. Therefore, when subjected to glycol oxidation facilitated by intrinsic defects and surface oxygen vacancies, Au/Co_3_O_4_ NL {111} exhibited much greater selectivity and catalytic activity than its Au/Co_3_O_4_ NC {001} counterpart (Fig. 9g). One potential catalyst for the oxidation of EG using Au NPs is Co_3_O_4_ NL {111}, which facilitates the activation of O_2_*via* the oxygen vacancies on its surface.

Furthermore, the catalysts Au/Co_3_O_4_ P were evaluated in the CO oxidation processes.^80^ The catalytic activity was substantially enhanced by adding Au NPs, as shown in Fig. 9h. This resulted in a noteworthy CO conversion of 35% at 20 °C and complete at 80 °C. As depicted in Fig. 9i, the activation energy (*E*_a_) for CO oxidation in Au/Co_3_O_4_ P is 15.49 kJ mol^−1^. Therefore, oxygen molecules follow the Langmuir–Hinshelwood mechanism, which catalyzes CO oxidation at low temperatures (20–60 °C) *via* Au/Co_3_O_4_ P {111}. In particular, rather than traversing the surface lattice oxygen sites, CO should be adsorbed onto oxygen vacancies at the surface and activated by Au NPs. The durability of the Au/Co_3_O_4_ P catalysts was also evaluated at temperatures of 25 and 60 °C (Fig. 9j). Throughout the twelve-hours CO oxidation process at 25 °C, the Au/Co_3_O_4_ P catalyst activity decreased from 2.92 to 1.87 mol_reactedCO_ g_Au_^−1^ s^−1^. A minimum activity of 5.26–5.39 mol_reactedCO_ g_Au_^−1^ s^−1^ was recorded for 9 h at 60 °C. This phenomenon might be primarily attributed to the surface oxygen vacancies and inherent defects of Co_3_O_4_ {111}, which activated O_2_. Similarly, the presence of Au^0^, Au^*δ*+^, and Au^+^ species on the surface of Au NPs further enhanced the activation of CO.

## 5. Chemical nature of the oxide particle morphology

Many people think that certain cobalt cations are abundant at the active sites. Co_3_O_4_ NR, rich in Co^3+^ cations and having mostly exposed {110} surfaces, is very active in low-temperature CO oxidation.^64^ Moreover, among Co_3_O_4_ NR, Co_3_O_4_ NC, and Co_3_O_4_ NP, Co_3_O_4_ NS with mostly exposed {111} planes enriched in Co^2+^ cations are the most active.^38^ At low temperatures, a Co_3_O_4_ SiO_2_ nanocomposite devoid of ordered planes but abundant in Co^2+^ proved an exceptionally active catalyst.^81^ However, these findings were mainly obtained *via* catalytic research, and direct spectroscopic evidence of the active surface oxidation state was absent.

**Fig. 10:**
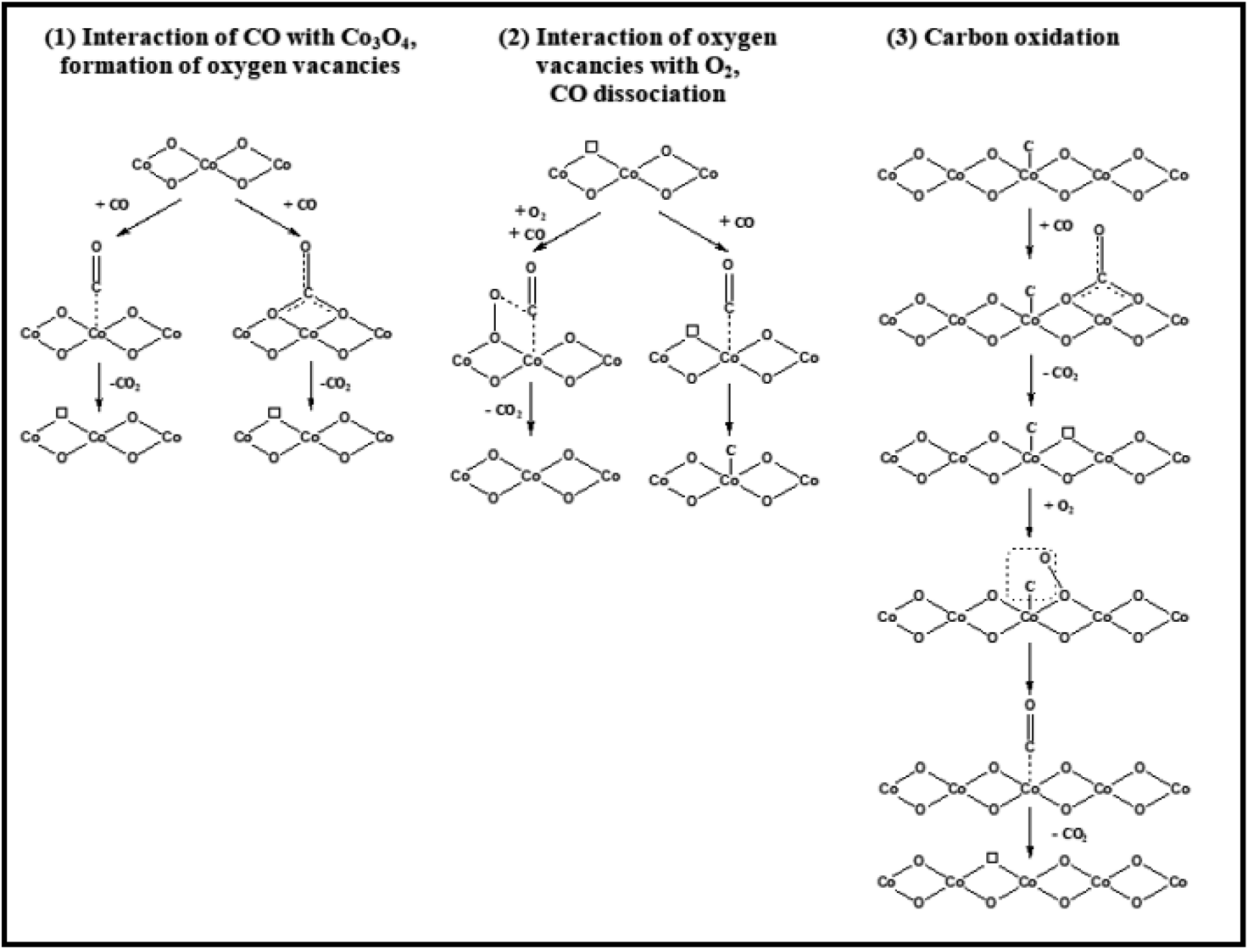
Schematic representation of CO oxidation on Co_3_O_4_. Reprinted with Permission from ref. 82, 2018 American Chemical Society.

Contrary to comparable nanostructures, there have been consistent findings on the shape influence of Co_3_O_4_ with nanometric size in catalyzing oxidation processes (as shown above). The many reaction routes can contribute, including changing the reaction conditions (primarily the gas and temperature). CO may be oxidized by the Langmuir–Hinshelwood method, which requires surface oxygen species, or the Mars-van Krevelen mechanism, which utilizes lattice oxygen species, according to spectroscopic observations^82^ and the spectroscopically examined possible reaction pathways/elementary steps of CO oxidation on Co_3_O_4_ are configured in Fig. 10, the former exhibited dominance at over 100 °C due to oxygen vacancy formation and the Co^3+^/Co^2+^ redox cycle. Conversely, at lower temperatures, the latter demonstrated dominance. One possible reaction mechanism is that CO adsorbs onto Co^3+^cations and then absorbs oxygen from the surface lattice coordinated to three Co^3+^ cations. The oxygen vacancy is then filled with oxygen from the gas phase, according to the Mars–van Krevelen mechanism.^64^

Spectroscopic evidence is lacking, although an interaction between molecularly adsorbed CO and O–O peroxo species has been postulated by analyzing the impact of pretreatment conditions,^65^ although no peroxo O–O species were found using *in situ* Raman spectroscopy.^81^ According to *in situ* infrared research, CO adsorbed on Co^2+^ sites interacted with an oxygen atom bound to a nearby Co^3+^ cation, and the gas phase oxygen was used to fill the oxygen vacancy.^83^ Isotopes are vital in the redox Mars–van Krevelen process and are responsible for CO oxidation.^84,85^

**Fig. 11:**
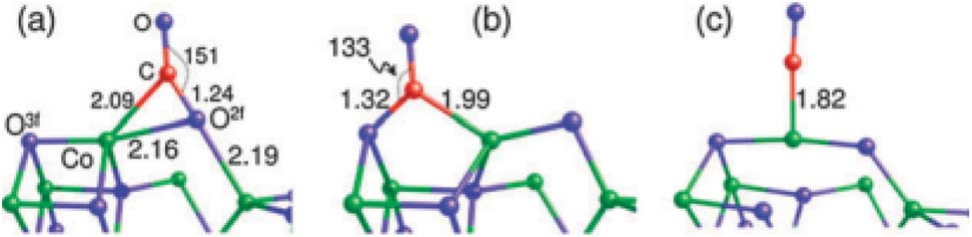
Three adsorption configurations of CO on Co_3_O_4_(110): (a) on O^2f^; (b) on O^3f^; (c) on Co. Bond lengths are in angstroms; bond angle are in degrees. Co, green, O, blue, and C, red. Reprinted with Permission from ref. 88, 2011 Royal Society of Chemistry.

Theoretical investigations into the CO oxidation pathway on Co_3_O_4_ have also shown differences.^86–89^ For instance, a Mars–van Krevelen process involving mostly exposed {110} planes in Co_3_O_4_ has been proposed, as shown in Fig. 11.^88^

**Fig. 12:**
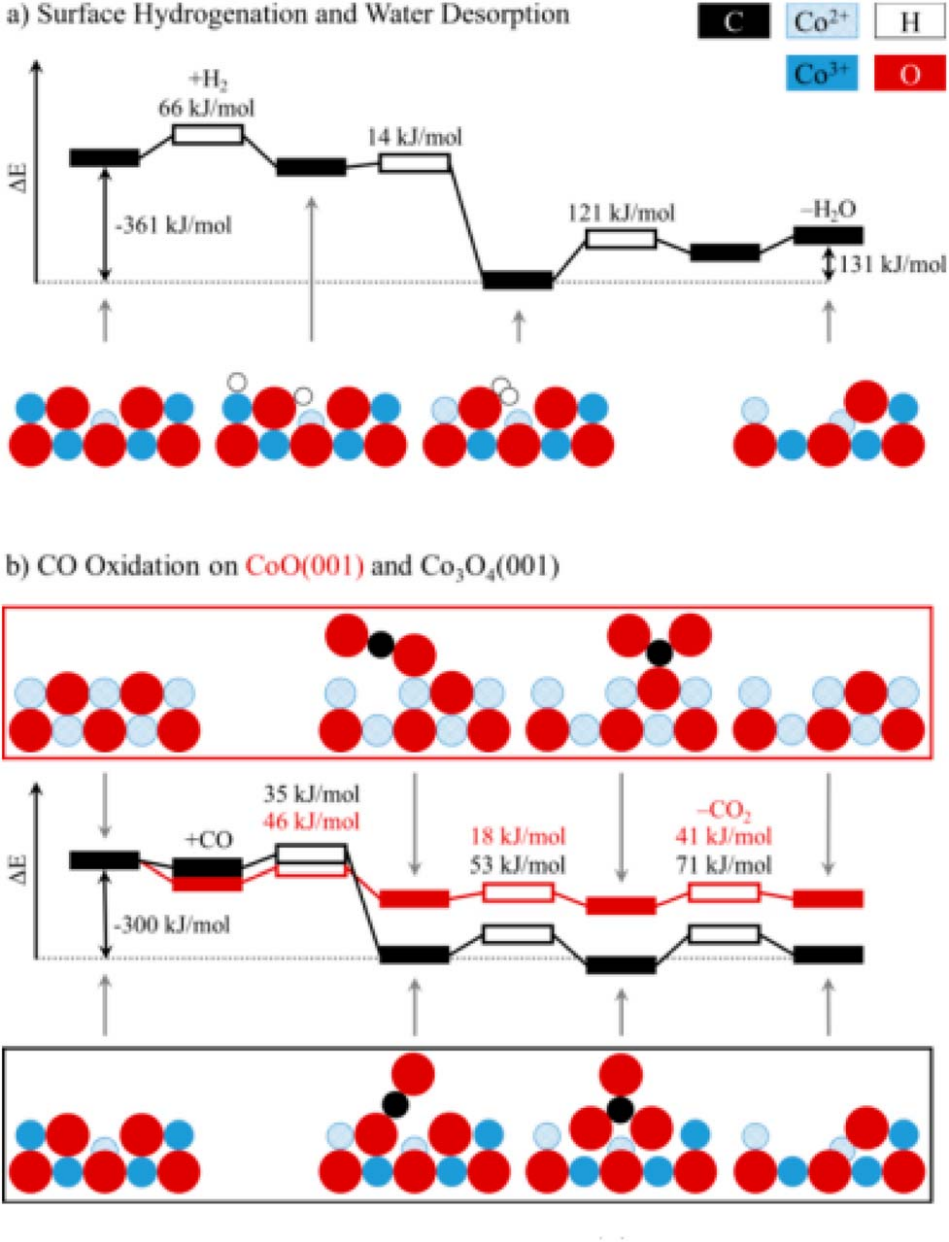
Potential energy diagrams for (a) the hydrogenation of Co_3_O_4_ {001} and (b) the oxidation of CO to CO_2_ on Co_3_O_4_ {001} and CoO {001}. For each transition state (hollow boxes), reaction barriers are given in kJ mol^−1^. Selected intermediates are shown as a side view along [110], using the following color codes: black (C), blue (Co), red (O), and white (H). Reprinted with Permission from ref. 90, 2019 American Chemical Society.

Theoretically, the octahedrally coordinated Co^2+^ site in CoO^90^ would be the most active site for the PROX of CO in the hydrogen-rich stream. According to DFT calculations, the generated carbonates should make the {001} facet of Co_3_O_4_ less reactive by blocking the surface sites on that facet but not on CoO {001}, as shown in Fig. 12.

Surface and lattice oxygen species interact concurrently in the reaction network, making methane oxidation on Co_3_O_4_ catalysts more difficult. There were three distinct temperature/conversion phases in the methane oxidation process, identified by the presence or absence of the adsorbed or lattice oxygen and the catalyst's redox state.^91^ At temperatures between 300 and 450 °C, the dominating superficial Langmuir–Hinshelwood structure produces a stoichiometric {100} surface on Co_3_O_4_ NC with a regular size of around 40–60 nm and with the preferential exposure {100}, as previously shown for CH_4_ combustion on these particles. At temperatures ranging from 450 to 650 °C, where O_2_ nearly occupies the oxygen vacancies generated by the emission of CO_2_ and H_2_O, the imperfect surface area is delineated by the coexistence of the interfacial (Mars–van Krevelen) and suprafacial (Langmuir–Hinshelwood) mechanisms.^92,93^ At temperatures over 650 °C and with a non-stoichiometric surface area, the completion of oxygen vacancies is only partial, resulting in a substantial reduction in catalyst activity and the combustion of CH_4_*via* the Mars–van Krevelen technique.^91^

**Fig. 13:**
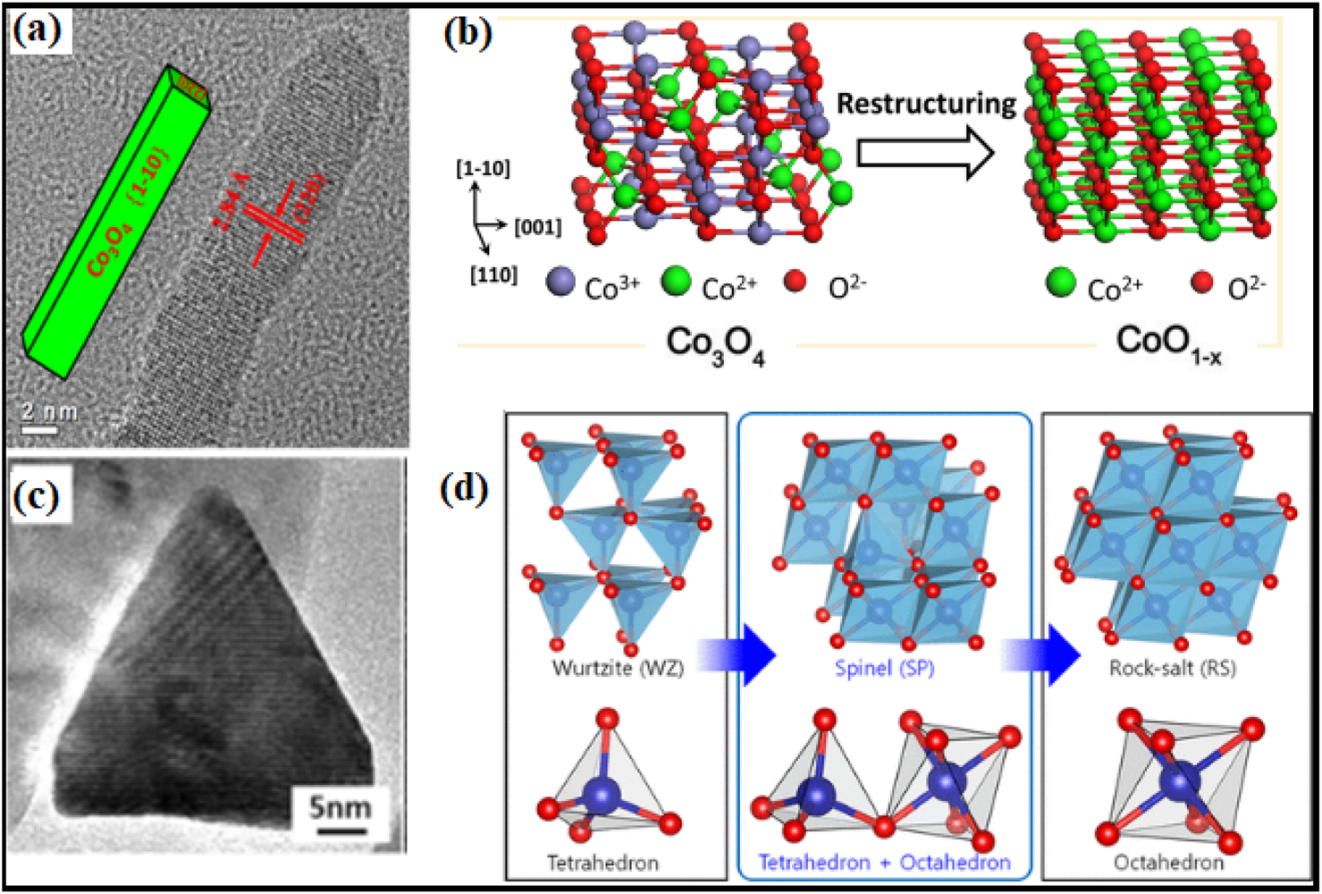
Structural transformation of Co_3_O_4_NR: (a) high-resolution transmission electron microscope (HRTEM) image; (b) schematic illustration of Co_3_O_4_ to CoO transformation under reaction conditions; (c) HRTEM image of CoO hexagonal pyramid; (d) illustration of the phase transformation from metastable wurtzite (WZ) CoO to stable rock-salt (RS) CoO *via* the intermediate spinel (SP) Co_3_O_4_. Reprinted with Permission from ref. 90 and 101 Copyrights 2013 and 2019, American Chemical Society.

Theoretical computations have led to the notion that the C–H bond in CH_4_ would be activated by the doubly coordinated lattice oxygen (O_2c_) across the {110} surface. Therefore, the {110} surface is expected to exhibit more activity than the {100} surface, devoid of any O_2c_ sites.^94^ Assuming dissociation of CH_4_ on the Co–O pair; researchers^95^ indicated that the reactivity of methane combustion increased as follows: {001} < {011} < {112}. Experimental observation of cubic Co_3_O_4_ revealed the less active {001} facet, while flower-shaped Co_3_O_4_ exhibited the active {111} facet.^96^ As compared to spherical NPs enclosed in the {001} and {111} facets or Co_3_O_4_ NRs exposed to the {110} and {001} facets, Co_3_O_4_ NLs encased in the {112} facet showed higher activity in the CH_4_ combustion process.^97^ The surface remodeling during reaction circumstances may contribute to the contradicting findings on the reactive facets. It has been shown by molecular modeling of Co_3_O_4_ NPs that the form may be maintained; however, when exposed to oxidizing and reducing atmospheres, the relative ratio of {111}/{100}/{110} facets changes dynamically.^98^ Under conditions rich in hydrogen gas, the faceting {110} plane was preferentially exposed. At the same time, the {111} surface remained untreated due to the development of oxygen surface vacancies and their subsequent diffusion toward the bulk. Nevertheless, the oxygen-rich conditions promoted the {111} termination. Therefore, it was necessary to describe the shape of the active catalysts. Recent breakthroughs in high-resolution microscopic and spectroscopic methods have opened the door to studying the functions of shaped-synchronized NPs in terms of their dynamic performance. Nitric oxide (NO) may be reduced with CO by reshaping Co_3_O_4_ NRs with an exposed {110} surface into non-stoichiometric CoO_1−*x*_ NR (Fig. 13a and b).^99^ The structure-modified NRs generate nitrogen gas by selectively reducing nitrogen oxides (NO_*x*_) with CO at temperatures ranging from 250 to 520 °C. Environmental transmission electron microscopy (ETEM) and ambient pressure X-ray photoelectron spectroscopy showed that the non-stoichiometric CoO_1−*x*_ NRs had a rock-salt (RS) structure. The 100% selectivity was brought about by the active phase, which included around 25% oxygen vacancies. Electron transport microscopy measurements in environments rich in hydrogen showed that CO_3_ was reduced to CO, indicating the formation of a boundary contact for particles larger than 15 nm but not for smaller ones, showing that smaller NPs undergo rapid reduction.^100^ ETEM identified a two-step phase transition during the heating experiment, as shown in Fig. 13c and d. In the low-temperature range of 200 to 280 °C, the wurtzite (WZ) CoO was spontaneously oxidized to spinel (SP) Co_3_O_4_ owing to the residual oxygen in the TEM. Secondly, under low oxygen partial pressure conditions, SP Co_3_O_4_ was reduced to RS CoO at temperatures reaching 280 °C.^101^ These visual results show that the as-prepared oxide NPs changed significantly under response conditions.

## 6. Concluding remarks and perspectives

Extensive exploration into the field of nanocatalysis utilizing Co_3_O_4_ nanometrics has undeniably demonstrated that the size and shape of the catalyst at the nanoscale level profoundly impact its catalytic effectiveness. A growing body of evidence suggests that the configuration of the nanometric Co_3_O_4_ is always critical in achieving optimal levels of selectivity, stability, and catalytic activity. This technology's advancement has been significant due to the incorporation of morphology-dependent nanocatalysts, an innovative tool for finely adjusting catalytically active sites. Both theoretical and experimental investigations have been extensive into the morphology-dependent nanocatalysis of nanometric Co_3_O_4_. Specifically, the arrangement of surface Co^3+^/Co^2+^ and O sites,^102–104^ focusing on the oxygen vacancy, has been linked to the catalytic properties of reactive surface facets. However, there are conflicting reports regarding the effectiveness of similar nanostructures in catalyzing different processes or even the same reaction under identical conditions. This suggests that the form-dependency of nanometric Co_3_O_4_, as documented, is highly susceptible to variations in reaction parameters and established reaction pathways.

The relationship between the catalytic activities of nanometric Co_3_O_4_ and the selectively exposed facets induced by shape has been demonstrated through experimental evidence. However, it cannot be ruled out that adjacent facets may work together synergistically. Initially designed nanostructures may undergo structure, morphology, and chemistry changes under actual reaction conditions. The catalytic properties observed in the experiments are determined by the dynamic behavior of the catalyst particles in response to temperature and the reactive environment rather than their state when prepared or recently used. In some instances, the activation of species in a multi-molecule chemical reaction may occur through diffusion on adjacent facets, resulting in a synergistic effect where the species activated by the adsorbed reactant can adsorb and stimulate a different type of reactant. *In situ* studies, physical and chemical analyses, and dynamic characterization techniques must be employed in operational environments to fully understand functional nanostructures.

To gain a deeper understanding of the relationships within nanostructured catalysts, further exploration is needed to develop improved experimental and theoretical methods.^105–107^ Variations in temperature and reactive gas fluctuations can impact the well-defined form of Co_3_O_4_ nanometric, leading to changes in its electrical and geometric properties. This, in turn, influences the proportion of active surfaces and the coordination environments of oxygen and cobalt atoms on the surface, ultimately affecting the development of active sites. The lack of published studies on the atomic structure of nanometric Co_3_O_4_ can be attributed to the limited availability of high-resolution spectroscopic and microscopic characterizations among researchers worldwide. Also, studying active sites' dynamic performance under operational conditions would provide valuable insights into the structure–reactivity relationship. By employing techniques that allow for real-time assessment of size, shape, interfacial structure, and gas-induced structural changes at the active sites of individual nanoparticles, combined with spectroscopic methods, we can significantly enhance our understanding of the inherent active regions and dynamic capabilities of nanostructured catalysts within catalytic environments.

## Abbreviation

BET: Brunauer–Emmett–Teller
CRR: CO_2_ reduction reaction
DFT: Density functional theory
EG: Ethylene glycol
MB: Methylene blue
NBs: Nanobelts
NCs: Nanocubes
NH: Polyhedron
NLs: Nanoplates
NPs: Nanoparticles
NRs: Nanorods
ORR: Oxygen reduction reaction
PLD: Pulsed laser deposition
PS: Persulfate
PROX: Preferred oxidation
SEM: Scanning electron microscopy
TEM: Transmission electron microscopy
XRD: X-ray diffraction

## Conflicts of interest

The authors declare that they have no known competing financial interests or personal relationships that could have appeared to influence the work reported in this paper.
